# Dynamic Vehicle Detection via the Use of Magnetic Field Sensors

**DOI:** 10.3390/s16010078

**Published:** 2016-01-19

**Authors:** Vytautas Markevicius, Dangirutis Navikas, Mindaugas Zilys, Darius Andriukaitis, Algimantas Valinevicius, Mindaugas Cepenas

**Affiliations:** Department of Electronics Engineering, Kaunas University of Technology, Studentu St. 50–418, LT-51368 Kaunas, Lithuania; dangirutis.navikas@ktu.lt (D.N.); mindaugas.zilys@ktu.lt (M.Z.); darius.andriukaitis@ktu.lt (D.A.); algimantas.valinevicius@ktu.lt (A.V.); mindaugas.cepenas@ktu.edu (M.C.)

**Keywords:** magnetic field, AMR sensors, vehicle speed detection

## Abstract

The vehicle detection process plays the key role in determining the success of intelligent transport management system solutions. The measurement of distortions of the Earth’s magnetic field using magnetic field sensors served as the basis for designing a solution aimed at vehicle detection. In accordance with the results obtained from research into process modeling and experimentally testing all the relevant hypotheses an algorithm for vehicle detection using the state criteria was proposed. Aiming to evaluate all of the possibilities, as well as pros and cons of the use of anisotropic magnetoresistance (AMR) sensors in the transport flow control process, we have performed a series of experiments with various vehicles (or different series) from several car manufacturers. A comparison of 12 selected methods, based on either the process of determining the peak signal values and their concurrence in time whilst calculating the delay, or by measuring the cross-correlation of these signals, was carried out. It was established that the relative error can be minimized via the Z component cross-correlation and K_z_ criterion cross-correlation methods. The average relative error of vehicle speed determination in the best case did not exceed 1.5% when the distance between sensors was set to 2 m.

## 1. Introduction

Intelligent transport [1], smart cities [2,3] and the Internet of Things [4] are terms that surround all of us nowadays. One of the key factors allowing one to consider intelligent solutions [3,4,5] specifically designed for urban areas is the constantly decreasing price of sensors capable of providing information about traffic flows, environmental conditions and other parameters. The processes of integrating sensors into the existing infrastructure and forming large sensor networks has thus become increasingly less complicated. In turn, the physical medium is less disrupted and society gains access to a brand new quality of life, facilitated by environmentally responsive public services, continual surveillance and a direct connection with other information users [6].

The capability to detect vehicles is the main element in intelligent transport management systems, allowing us to gather information about the traffic intensity and vehicle speed. Amongst the most popular transport sensors are induction loops, commonly used due to their low price and relatively high signal quality. However, the process of installing such devices normally results in significant damage to an extensive area of the road surface and the loop itself may suffer from the thermal expansion of the road pavement. Anisotropic magnetoresistance (AMR) sensors are an alternative to induction loops capable of measuring even extremely weak magnetic fields. When a vehicle is above the sensor, its metal construction distorts the Earth’s magnetic field and this allows one to determine the location of the vehicle.

Magnetoresistive sensors are small, so their installation and maintenance are much less complex and pricey in comparison to induction loops. Nevertheless, since their introduction, they have been subject to little scrutiny or testing in this particular application area.

As the Earth’s magnetic field distortion signal can be used not only for the detection, but also for the classification and recognition of transport vehicles [7], it is highly important to ensure sensor effectiveness and the repetition rate of the recognized signal. Various objects and processes taking place in the surrounding environment have a major impact on the readings of magnetic sensors. The factors determining the success of locating a vehicle are multiple, therefore, distinguishing the distortions of the magnetic field caused by the vehicle from those caused by certain environmental changes is a rather difficult task [8,9]. The accuracy of locating vehicles in the environment with no undesirable objects affecting the magnetic field would be significantly higher, but, unfortunately, the possibilities of creating such an environment are slim to none at all. Taking that into account, in comparison to road induction loops the process of locating vehicles using magnetic fields is way more complicated. When conducting the task of locating a vehicle using the magnetic field method, these external factors must be eliminated. The literature analysis reveals that the majority of research in the field of AMR sensors has been carried out in laboratories, which indicates the lack of practical research activity when it comes to real transport vehicles and road environment conditions [10].

## 2. Related Works

Previous research papers mostly addressed the problems related to the detection of vehicles parked on parking lots or in car parks [11]. The measurement system based on AMR sensors was thus created for the purpose of locating vehicles using the Earth’s magnetic field distortion method. It comprises magnetoresistive sensors produced by four different manufacturers and researchers have focused on analyzing the temperature stability and parameter spread influence on detection relevant to reliability [12]. Following a lengthy period of observation, the researchers determined several shortcomings of the method, which were as follows: dependence of the sensor signal on temperature, residual magnetic moment and diffusion of the sensor parameters during the manufacturing process [13]. Upon analyzing the impact of the vehicle construction on the magnetic field changes in the parameters of the mounted sensors, it was determined that said construction plays a vital role in terms of magnetic field distortion and sensor output signals. Even vehicles of similar construction impact the magnetic field components differently when entering and exiting a parking space. The amplitude and form of an AMR sensor signal is also dependent on how the sensors and vehicles are arranged in terms of their positions magnetic fieldwise [13]. When a vehicle is on top of the sensor, in some cases one can determine the critical zones which display no change in the magnetic field. Based on modeling and practical research results, researchers have proposed a location algorithm reliant on the state criteria. It has been established that the most accurate way of determining the state of the parking location sensors relies on the use of complex criteria [14]. Via the use of this method the researchers observed a 93.3% detection effectiveness.

In the case of moving vehicles, the majority of the proposed stationary vehicle detection methods have been proven unsuitable due to the need to not only record the fact of the vehicle’s presence, but also measure its speed and the dependence of the magnetic field distortions on the vehicle construction. It is noteworthy that the majority of publications contain algorithms and methods designed to locate vehicles and determine their speed via the use of AMR sensors, yet most of these papers lack any consideration of the fact that small sensors can be passed by vehicles following different trajectories, which can significantly complicate the task of determining the vehicle type.

The bottom of the vehicle under the engine is commonly magnetically open whilst the engine section holds a generator and the ignition starter, each of which generate their own operating magnetic fields. In between the engine department and vehicle interior there is typically a steel (magnetic) partition, similar to the ones found between multiple interior and baggage compartments. Much of uncertainty is caused by the aforementioned partitions, knowing that regardless of their distance to the AMR sensor, they can act as a vehicle driving nearby, or the one being located above, *i.e.*, the sum of the magnetic field and its separate components, subject to a partition in the vicinity of the magneto-resistive sensors, can increase or decrease. Therefore, the notion that the Z component of the Earth’s magnetic field increases under the vehicle is not necessarily true in all cases, and this can significantly complicate the process of identifying a vehicle located in the same line as the sensor.

## 3. Experiments

**Figure 1:**
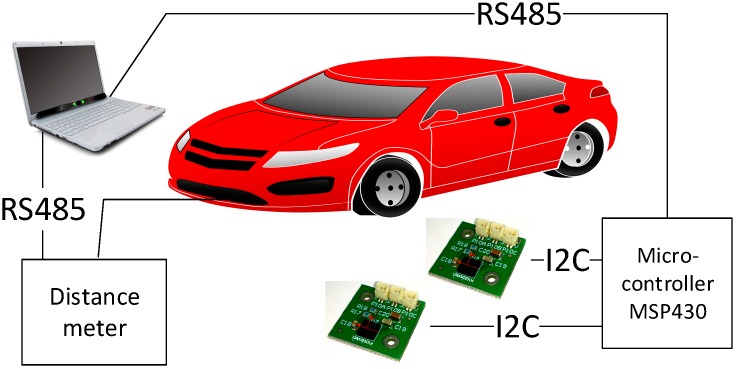
Experiment structure.

**Figure 2:**
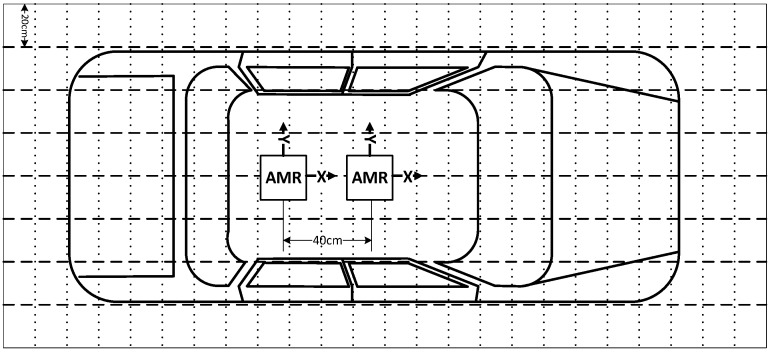
Vehicle scanning scheme.

An experiment was conducted with 24 various models of cars produced by different manufacturers seeking to evaluate the possibilities as well as pros and cons of using AMR sensors in the control of transport flows. A brand new vehicle scanning system (Figure 1) was designed, which allowed us to measure the distortion of the Earth’s magnetic field caused by vehicles. Vehicle detection was conducted using two sensors located at a distance of 30 cm. The measurement was carried out every 1 cm along the X axis direction whilst capturing the vehicle position and the magnetic field parameters. Scanning along the direction of the X axis was repeated every 20 cm along the direction of the Y axis whilst moving both AMR sensors (Figure 2).

For the purpose of gathering data a digital distance measuring instrument was used, which transferred all the data via a RS 485 serial communication port to a computer where it was then processed with the specifically designed software. The data acquired from the sensor via the RS 485 port. The process of transferring data and reading the data from the AMR sensors via the I2C interface was carried out using a low-power MSP430 microcontroller.

**Figure 3:**
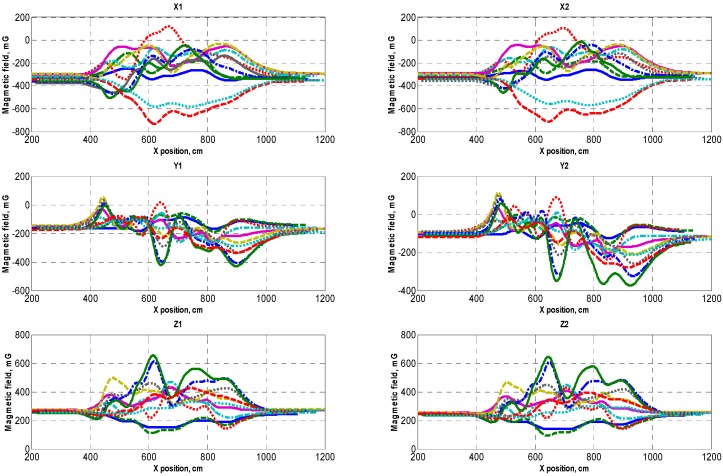
The distribution of the magnetic field measured by two distinct sensors (plots of magnetic field components X1, Y1, Z1 and X2, Y2, Z2 of sensors 1 and 2 respectively). Plot curves are color coded by sensor position along latitudinal vehicle line.

The Earth’s magnetic field distortions were detected on different vehicles. A sample set of the Earth’s magnetic field distortions data caused by several of them is provided in Figure 3. The research results indicate that the change in the magnetic field modulus and separate components when the vehicle is driven on top of the magneto-resistive sensor is different and depends on the vehicle brand and the position of sensors with respect to a particular vehicle (Figure 3). This is the reason behind the fact that when a vehicle passes the sensor in different places (with respect to the Y axis), very diverse magnetic field profiles are recorded. As a result, the methods based on measuring and comparing absolute values are unsuitable for detecting the type and speed of vehicles.

**Figure 4:**
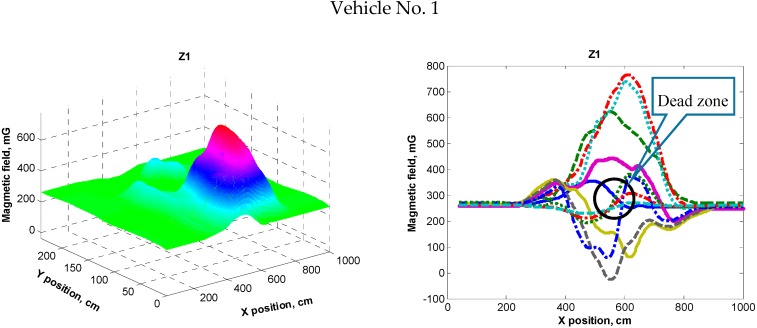

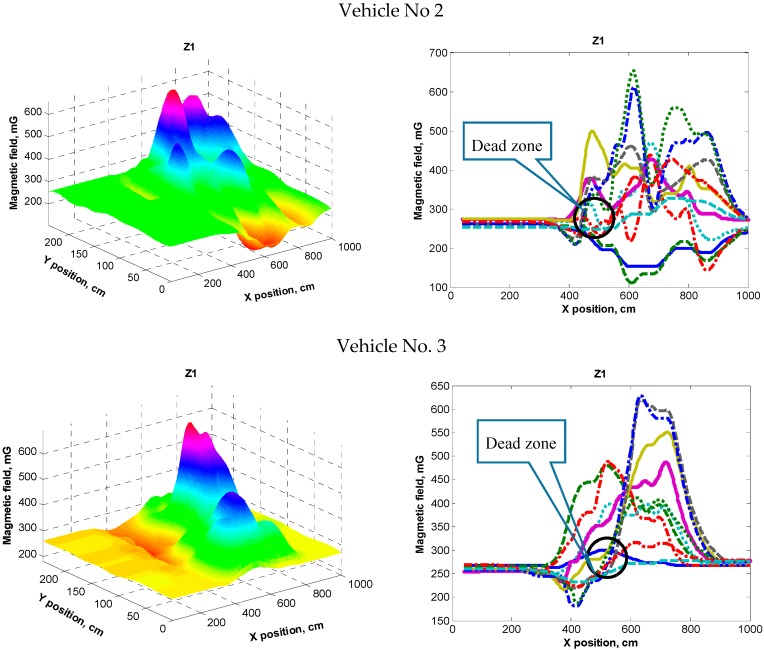
The distribution of the magnetic field distortion (Z component) caused by three different vehicles (different color means different position of sensor with respect to the Y axis—across the vehicle).

Upon completing the interpolation of the acquired data, a full view of the magnetic field distortion caused by a vehicle was acquired (Figure 4). From the data it can be noticed that there are a number of areas (marked as “Dead zone”) under the vehicle where the magnetic field remains undistorted (as if the vehicle was not present at all). Such situations are critical when using AMR sensors for determining the presence of a vehicle in a static state.

## 4. Analysis of Vehicle Detection Methods

The key task which must be addressed when designing the system for detecting speed and vehicle type is related to the accuracy of determining the duration between signals from separate sensors. Once established the stable signal discretization frequency and completing the matching of both signals, it becomes possible to calculate the delay between signals and the vehicle speed.

Seeking to evaluate the accuracy of determining the vehicle speed using AMR sensors, we turned to the data acquired from conducting the experiment and carried out a comparison of 12 selected methods. The list of these methods is provided in Table 1.

**Table 1:** List of methods.

No.	Method
1	Z component peak detection
2	Z component cross-correlation
3	Module peak detection
4	Module cross-correlation
5	Vectorial deviation peaks (Equation (1))
6	Vectorial deviation cross-correlation (Equation (1))
7	Combined vectorial deviation—peaks (Equation (2))
8	Combined vectorial deviation cross-correlation (Equation (2))
9	Kz criterion peaks
10	Kz criterion cross-correlation
11	Z peaks ±50 readings of cross-correlation
12	Module peaks ±50 readings of cross-correlation

The selected methods are based on either the process of finding the peak signal values or their superposition in time whilst calculating the delay, or via calculation of the cross-correlation of these signals and determining the lag.

*Vectorial deviation* is a simple criterion defined as [11]:

(1) $$K=|\cos\alpha -\cos\alpha_{0}\left|\;+\;\right|\cos\beta -\cos\beta_{0}\left|\;+\;\right|cos\gamma -\cos\gamma_{0}|$$

where *α, β, γ*—angle between magnetic field vector and x, y, z axis respectively.

*Combined vectorial deviation* is a criterion compiled from the “square” and vectorial deviations. According to the fact that the Z component increases when a vehicle is moving over and decreases nearby the vehicle, it can be used to increase the sensor’s sensitivity when a vehicle is moving:

(2) $$K=|\cos\alpha -\cos\alpha_{0}\left|\;+\;\right|\cos\beta -\cos\beta_{0}|\;+\;(B_{z}/B_{z0}-1)$$

where cos *α*, cos *β*—cosines of magnetic field vector’s angles on the influence of vehicle, cos *α*_0_, cos *β*_0_—cosines of magnetic field vector angles without influence of vehicle, $B_{z}$—magnetic induction on the influence of vehicle, $B_{z0}$—magnetic induction value without influence of vehicle.

*K_z_* criterion:

(3) $$K_{z}=\frac{B_{z}}{B_{z0}}-1$$

where $B_{z0}$—magnetic induction value without influence of vehicle.

**Figure 5:**
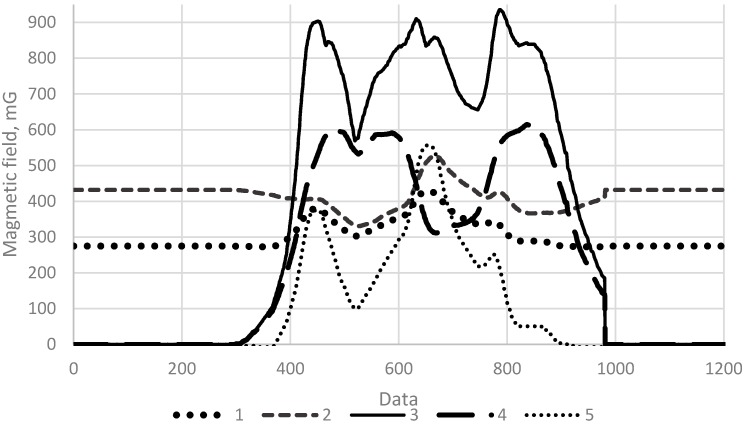
The gathered data when using different methods (1—Z component, 2—Module, 3—Vectorial deviation, 4—Combined vectorial deviation, 5—*K_z_* criterium).

The view of the gathered data when using different methods which are interpreted in search for signal matching, is presented in Figure 5.

**Figure 6:**
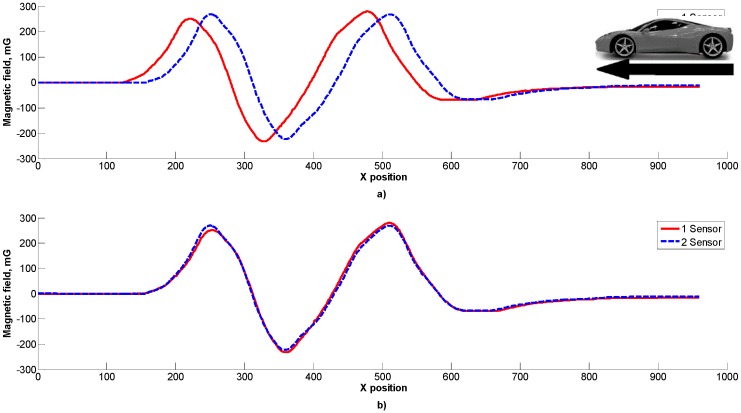
A view of the data samples from two sensors (**a**) and samples matching (**b**) via the use of the Z component.

In Figure 6 a view of the data samples from two matching sensors via the use of Z component cross-correlation is presented. The mismatch between data samples from the two sensors could be due to the different sensitivity or positioning of sensors. Such a mismatch could be eliminated by using data normalization techniques.

Upon conducting the analysis and processing of the data collected during the experiment using the selected signal matching methods, we have determined the average and maximum relative errors when the distance between sensors was considered to be equal to 2 m. The results are displayed in Table 2.

**Figure 7:**
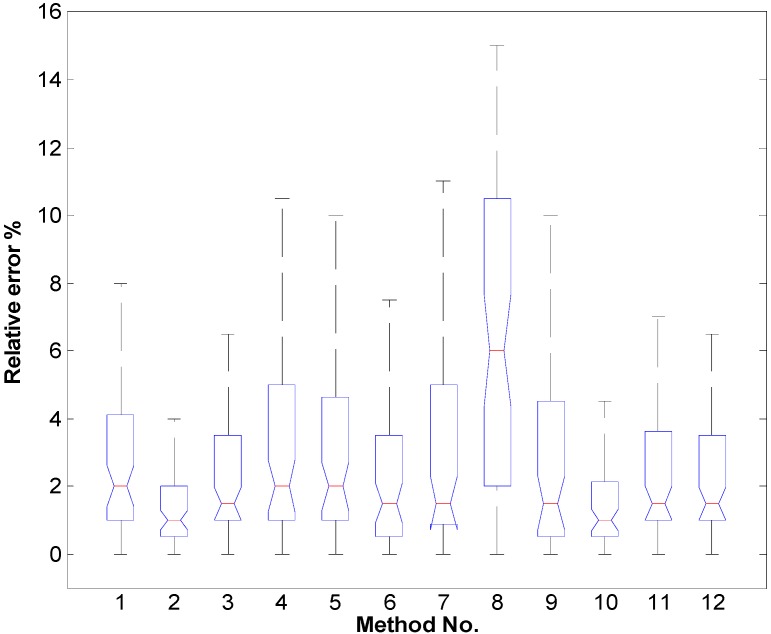
Box plot of the methods’ (Table 1) relative errors.

**Figure 8:**
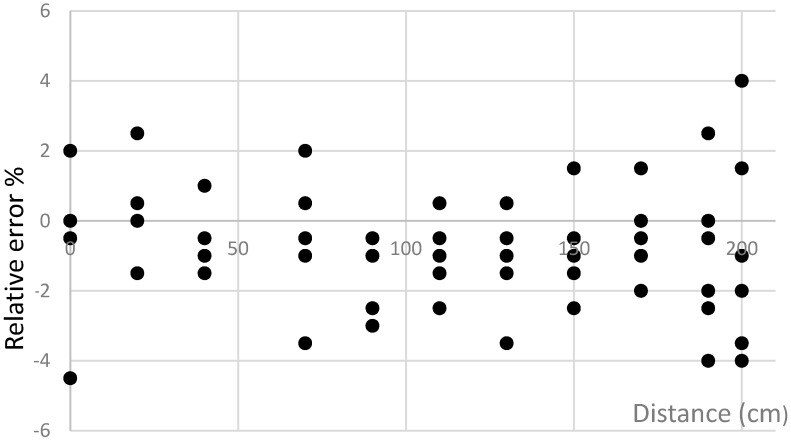
The dependence of error on the position of sensors with respect to vehicles using the second method.

**Figure 9:**
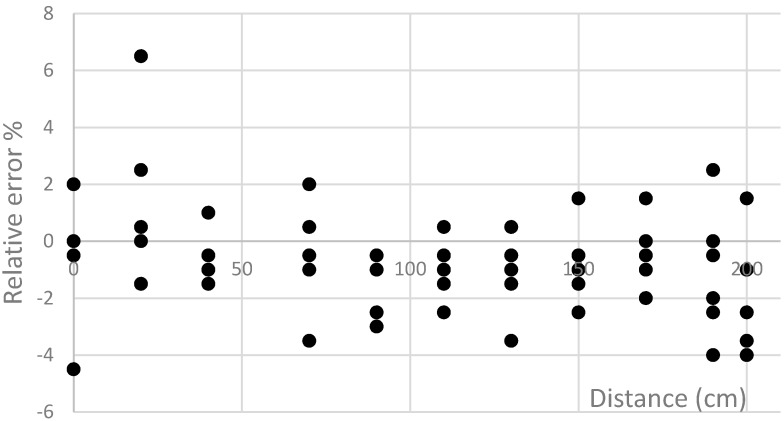
The dependence of error on the position of sensors with respect to vehicles using the tenth method.

The analysis of the acquired results has revealed that the smallest average relative errors are obtained with the second and the tenth methods (the maximum errors do not exceed 6.5%, Figure 7). The dispersion of errors in the case of using these methods is shown in Figure 8 and Figure 9.

**Table 2:** Method errors.

Relative Error (%)	Method No.											
	1	2	3	4	5	6	7	8	9	10	11	12
Average	3.6	1.5	2.8	3.3	3.7	6.7	3.3	2.6	3.9	1.5	3.1	2.5
Maximum	28.5	6.0	14.5	15.0	15.0	23.5	12.5	12.5	31.0	6.5	15.5	11.0

Sensor placement near the longitudinal centerline of the passing vehicle (Figure 8 and Figure 9) increases the precision of the speed detection. The analysis of the obtained results indicates that if the right signal matching method of AMR data samples is selected, it is possible (with an acceptable error) to determine the speed and type of the vehicle (considering the length of vehicle). The benefits of such a system are as follows:

1. The detection of the vehicle location, measurement of speed and classification of vehicles according to their type.
2. Lower infrastructure costs.
3. Relatively modest amount of data to be transferred due to the fact that signal processing can be performed in the sensor itself.

## 5. Ideas for Further Research

There is great potential for further research into the use of AMR sensors in the presence of a different Z component angles (*i.e*. at different locations across the Earth) and for analyzing different types of signal processing methods for vehicle speed detection. Moreover, there is still a knowledge gap in terms of researching vehicle detection using the magnetic vehicle signature features.

## 6. Conclusions

By experimental research it has been established that AMR sensors can be used for the purposes of vehicle speed control and vehicle classification. Upon conducting research with regard to the different impact of distinct vehicle types on the Earth’s magnetic field it has been established that different vehicles have distinct magnetic signatures which can be used for identifying the vehicle type.

According to the research results it can be concluded that is sufficient to use only one AMR Z component for vehicle speed detection. Upon analyzing the 12 methods found suitable for the purpose of speed control, it has been established that the minimum errors are obtained using the Z component for methods 2 and 10 (the Z component cross-correlation and K_z_ cross-correlation criterion methods). When using the proposed methods the average relative error of speed determinations in the case of applying the most suitable method does not exceed 1.5% when the distance between sensors is 2 m. It should be noted however that the vehicles can affect measurements by adding additional errors. These can be eliminated through other magnetic field components.
